# Dr. J. C. Boardman’s Provings and Clinical Cases

**Published:** 1890-12

**Authors:** E. W. Berridge

**Affiliations:** London, England


### DR. J. C. BOARDMAN'S PROVINGS AND CLINICAL CASES.

E. W. Berridge, M. D., London, England.

The following are extracted from communications sent by Dr. Boardman to Dr. Swan, and from him transferred to myself. They are dated 1882, 1883.

(1) *Fluoric Acid.* Mrs. ——, aged about sixty, had complained for many years of one very annoying symptom, an aching pain *in one unvarying spot* in right side, just below diaphragm, on upper portion of liver; it was never *very* severe, but always present more or less. The symptom, "pain in a small spot," led me to give *Fluoric acid*$^{500}$ (Bœricke). In a few days the patient was well.

I find great use for *Fluor-acid;* so many cases of varied forms of disease do I meet, when there is some one spot in the body that will not yield, even when all other signs of disease disappear. I now give *Fluor-acid* $^{cm}$ (Swan) in all such cases, and always relieve, and often effect a complete cure in a few hours.

I have been prescribing for a gentleman now in his seventieth year who has suffered much from his heart, almost from childhood; sometimes *violent* palpitation, sometimes a *severe* aching, always an aching in one spot, though sometimes for days or weeks it would only slightly annoy. A few days ago he had the *severe* aching in the one spot, but no palpitation. I gave him one dose of *Fluoric acid*, and he felt a sense of relief in a manner he had never felt before.

The following is a proving: Mrs. ——, aged twenty-two, in excellent health, took one dose of *Fluoric acid*$^{500}$ (Bœricke) at four P. M., December 17th, 1883. In fifteen minutes a sharp pain passed across forehead, over eyes, and then passed directly down from middle of left clavicle into heart, cutting its way like a sharp knife. The suffering for the moment was terrific, but it soon passed off. Just before retiring, at half past nine P. M., a slight aching pain in nape. Her sleep was often interrupted, but whenever she woke she felt a heavy dull, aching, throbbing pain extending from nape over *whole* head.

December 18th.—On rising this morning, eyeballs felt sore and weak; eyes were at first pained by the light, and whenever she turned her eyeballs the soreness was very much aggravated. There was also some lachrymation. The whole head felt sore internally and externally. The roots of hair felt very sore on combing. The whole head felt compressed as if clasped.

At half past two P. M. all the above symptoms are gradually diminishing.

Quarter past three P. M.—All day has felt low-spirited, at times almost ready to burst into tears, and physically she is obliged to make great efforts to keep about.

On inquiring the exact direction of the pain across forehead, she said she could not positively say which it was. It came so suddenly and left so quickly, and seemed for the moment to have blinded her, that she cannot remember the direction; but inasmuch as the pain went down from the left clavicle, she concludes that the direction across forehead was from right to left. What she means by saying that the pain seemed for the moment to have blinded her is that consciousness for the moment had left her in fact, a *mental* blindness; her consciousness returned at the moment the pain was descending from middle of clavicle.

December 26th.—Had no further symptoms after 18th till to-day. About four P. M. to-day, was suddenly seized with sharp, piercing pains in heart, as though made by a small sharp penknife, moving from right to left, directly through the heart. These pains continued till seven P. M., more or less violent, when they gradually passed away. She retired about nine P. M., when for the first time she failed to hear the clear, loud tones of the door-bell, though at other times her hearing is excellent, always hearing this bell, in whatever part of the house she is. At that very time of not hearing the bell, she felt strange, felt lost, as though she did not realize what she was doing or should do; also her heart was in great distress, really indescribable. She seemed in a daze as to what was going on outside of herself; in fact, her perceptions of external things seemed in a great degree to have passed away.

December 27th.—Slept well, and had no further symptoms.

(2) *Triticum* (wheat) in Swan's potencies postpones the menses. Compare Swan's provings in *Organon*, vol. III, p. 114.

(3) *Alcohol.* I have never failed in a single instance of curing alcoholism by a high potency of Alcohol, even when the patients were unaware of what the remedy was. One of them began to drink lager beer three months after being cured, but he still had an aversion to whiskey.

(4) *Petroleum.* I have found this remedy in high potency the chief remedy for the annoying dribbling of urine to which so many very old men are subject.

(5) *Lager Beer.* On June 27th, 1883, at three P. M., I gave a lady, aged about twenty-five, one dose of *Lager Beer* $^{mm}$ (Swan), in order to cure her of an undue fondness for it.

She was never intoxicated, but used to drink a great deal of lager to keep up her strength to do hard work. In about thirty minutes she came bounding into my office like a mad woman. She was evidently in extreme suffering, and kept calling out again and again, "What have you given me?" I asked her symptoms, and while dancing up and down my office, she managed to say, "Very soon after taking the powder I had a desire to urinate. I have now a raging furnace flame in the entire urinary apparatus; a fierce urging, pressive burning desire to urinate constantly, so great that I am in perfect torture, but it only passes in drops." Very gradually the pains lessened.

Next day she had the same symptoms, but milder; and by the evening they all ceased and did not return. Since then her appetite, spirits, and strength have decidedly improved, and she has no desire for lager beer, and does not drink it at all. Never had similar symptoms before.

[NOTE.—This is a more complete version of the case published in THE HOMŒOPATHIC PHYSICIAN, 1884, p. 88.]

In the other cases published there, the following corrections and additions should be made:

Page 88, line five from bottom, for sixty-five read seventy-six.

Page 89, line two, for "chest" read "upper chest."

Page 89, line ten, for "great lameness" read "great aching pain and lameness."

This patient reported himself still quite well on December 4th, 1883.

The first case (p. 89, lines two to seven from bottom), which is reported "very much better," reported later "quite cured."

Dr. Swan wrote me April 9th, 1886, "I have successfully used *Lager* in high potency, in several other cases, where there was great heat in vesical region." This remedy deserves a further proving, but as I suppose Lager varies in composition, the original preparation used by Dr. Swan should be used.

